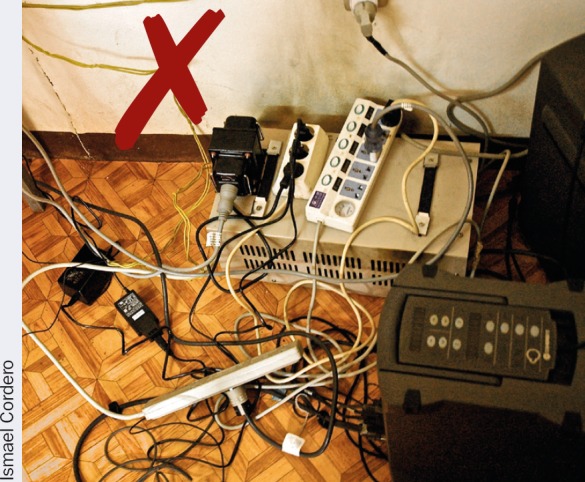# Electrical safety in the clinical environment – good habits to maintain

**Published:** 2015

**Authors:** Ismael Cordero

**Affiliations:** Clinical Engineer, Philadelphia, USA. ismaelcordero@me.com

**Figure:**
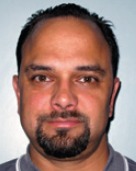
Ismael Cordero

There are many habits that clinical personnel can practise to help ensure an electrically safe environment. Here are twenty of these habits.

## Cords and plugs

**Figure 1:**
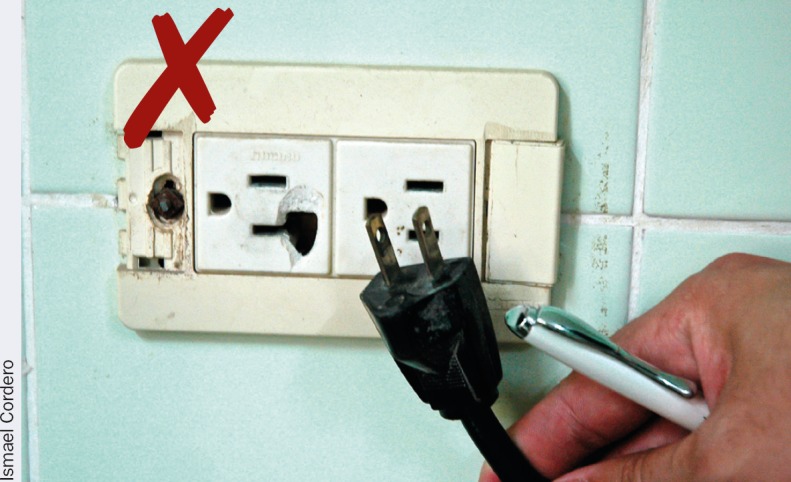


**Figure 2:**
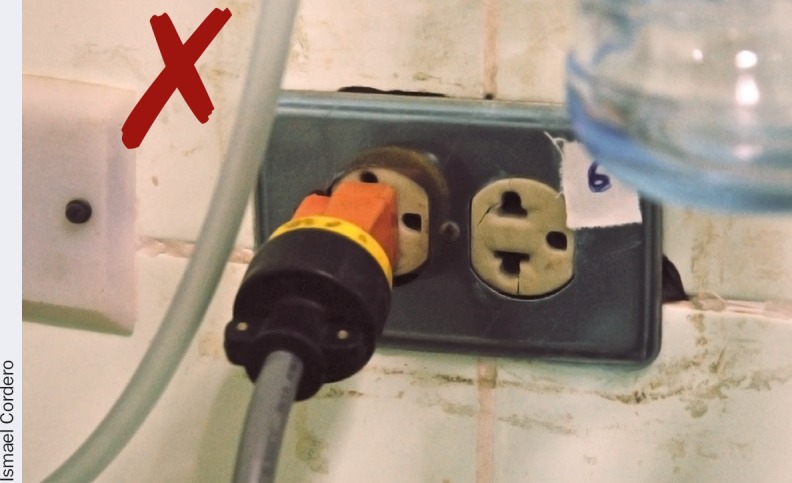


**Figure 3:**
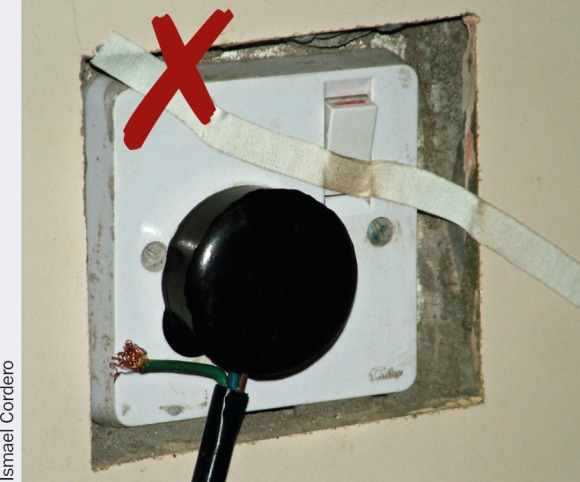


**Figure 4:**
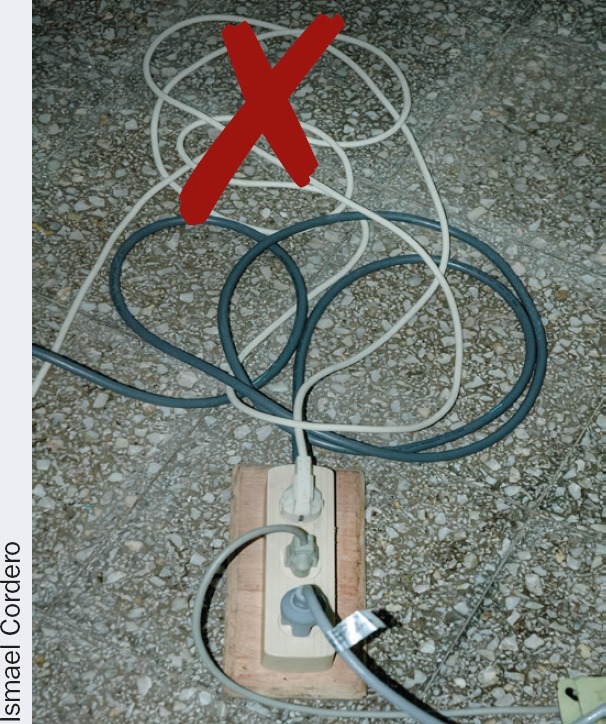


1. Only use electrical devices with 3-conductor power cords and 3-prong plugs (Figure 1 shows a plug with a broken ground prong – do not use).
2. Do not use cheater plugs (3-prong to 2-prong adapters, Figure 2). They eliminate the ground connection and increase the possibility of serious shock hazards.
3. Always unplug equipment by grasping the plug, not the cord.
4. Routinely check equipment power cords for frayed, cracked, or exposed wiring (Figure 3 shows a plug with the ground wire pulled out, which is very dangerous).
5. Do not rest cords over hot or sharp objects.
6. Do not run cords where they may cause a tripping hazard (Figure 4).
7. Avoid rolling equipment over equipment cords.

## Wall receptacles

8 Do not plug equipment into defective receptacles.
9 Plug equipment into wall receptacles with power switches in the OFF position.
10 Avoid using extension cords and power bars.
11 Do **not** overload electrical outlets by plugging in devices exceeding the current limit for the circuit (Figure 5).

## Fuses

12 Replace fuses only with the same exact type (voltage, amperes, slow-blow vs. fast blow, physical size). If the fuse of the correct rating is not readily available, and if the instrument has to be used in an emergency situation, a fuse of a lower rating can be used while waiting for the fuse of the correct rating. For instance, if a 250 mA fuse is required and is not available, the instrument will work with a 200 mA fuse if it is available. Fuses are usually over-rated for additional safety.
13 Do not continue to replace fuses if they keep burning out. Whatever is causing this must be found and corrected.

## General

14 Make sure your hospital engineering department performs regular safety and performance inspections on all equipment and electrical outlets.
15 Do not attempt to perform repairs yourself. A little knowledge can be a dangerous thing. Call your qualified biomedical equipment technician, the manufacturer or someone with technical troubleshooting and repair skills.
16 Make contingency plans for power failures.
17 If you suspect a fault, report it immediately to your engineering department. Never assume that someone else will take care of it.
18 Report any electrical tingling sensations promptly.
19 Keep equipment dry unless it is purposely designed to be wet.
20 Wear appropriately insulated shoes in wet areas.

**Figure 5:**